# Melanoma Metabolism: Molecular Mechanisms and Therapeutic Implications in Cutaneous Oncology

**DOI:** 10.1002/cam4.70386

**Published:** 2024-11-04

**Authors:** Isabella J. Tan, Aarushi K. Parikh, Bernard A. Cohen

**Affiliations:** ^1^ Rutgers Robert Wood Johnson Medical School New Brunswick New Jersey USA; ^2^ Department of Dermatology The Johns Hopkins Hospital Baltimore Maryland USA

**Keywords:** epigenetics, melanoma, metabolic reprogramming, metabolism, Warburg effect

## Abstract

**Background:**

Melanoma, a highly aggressive skin cancer, is characterized by rapid progression and a high metastatic potential, presenting significant challenges in clinical oncology. A critical aspect of melanoma biology is its metabolic reprogramming, which supports tumor growth, survival, and therapeutic resistance.

**Objective:**

This review aims to explore the key molecular mechanisms driving metabolic alterations in melanoma and their implications for developing therapeutic strategies.

**Methods:**

A Pubmed search was conducted to analyze literature discussing key mechanisms of the Warburg effect, mitochondrial dysfunction, enhanced lipid metabolism, epigenetic modifications, and the tumor microenvironment.

**Results:**

Metabolic reprogramming supports melanoma growth, proliferation, and survival. Understanding these complex metabolic dynamics provides valuable insights for developing targeted therapeutic strategies.

**Conclusion:**

Potential therapeutic interventions aimed at disrupting melanoma metabolism highlight the promise of precision medicine in improving treatment outcomes in cutaneous oncology. By targeting metabolic vulnerabilities, novel treatment approaches could significantly enhance the clinical management and prognosis of melanoma.

## Graphical abstract

A critical aspect of melanoma biology is its metabolic reprogramming, which supports tumor growth, survival, and therapeutic resistance. This review explores the key molecular mechanisms driving metabolic alterations in melanoma, including the Warburg effect, mitochondrial dysfunction, and enhanced lipid metabolism.

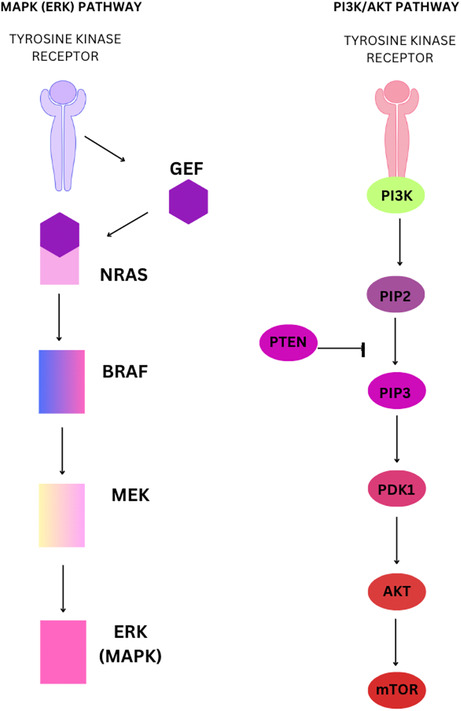

## 1 Introduction

**FIGURE 1:**
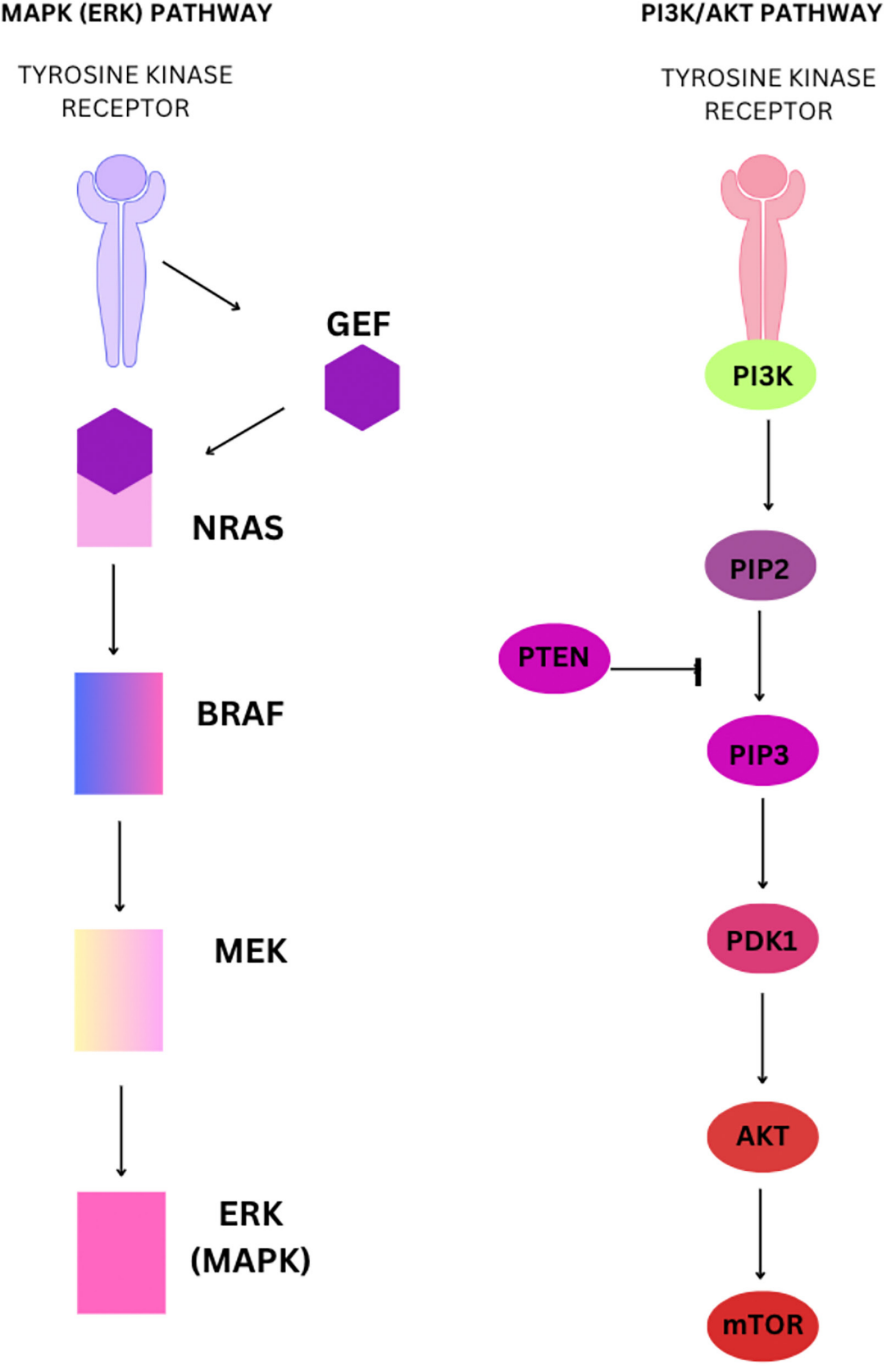
Signaling pathways implicated in melanoma cell growth, proliferation, and survival.

Cellular metabolism is foundational to biological processes, governing energy production, biosynthesis, and cellular signaling essential for growth and response to environmental cues [1]. In aggressive cancers like melanoma, metabolic reprogramming plays a crucial role, facilitating uncontrolled proliferation and survival by reshaping energy utilization and nutrient acquisition [2]. This shift includes increased glucose uptake, mitochondrial alterations, and changes in lipid and amino acid metabolism, supporting tumor growth and enhancing resilience under stress conditions. These adaptations, driven by oncogenic mutations and the tumor microenvironment, confer survival advantages, promoting invasion, immune evasion, and therapy resistance [2]. Understanding these metabolic dynamics is critical for developing targeted therapies that disrupt specific pathways, potentially improving outcomes in melanoma treatment through precision oncology approaches tailored to individual tumor profiles (Figure 1).

## 2 Methods

This literature review on melanoma metabolism and its therapeutic implications in cutaneous oncology involved a systematic search of peer‐reviewed articles and relevant databases. We utilized a comprehensive search strategy to identify studies focusing on molecular mechanisms, metabolic alterations, and therapeutic targets in melanoma. The screening for inclusion was conducted independently by two reviewers (I.J.T. and A.K.P.). Data from selected articles was utilized to highlight key findings, including metabolic reprogramming, regulatory mechanisms, and therapeutic strategies. Quality assessment was performed to ensure the reliability of the utilized evidence. While acknowledging potential limitations, including publication bias and variability in study methodologies, this review provides insights into melanoma biology and informs the development of novel therapeutic approaches targeting metabolic vulnerabilities in this malignancy.

## 3 Discussion

### 3.1 Overview of Metabolic Alterations in Melanoma

Within cutaneous oncology, melanoma distinguishes itself with intricate metabolic adaptations crucial for tumor progression and therapy responses. Melanoma cells prominently exhibit the Warburg effect, characterized by heightened glucose uptake and utilization, even under normal oxygen levels [3]. This metabolic shift drives increased glycolytic activity, generating pyruvate and lactate that contribute to the acidic tumor microenvironment [3]. Metastatic melanomas thus may rely more heavily on the tricarboxylic acid (TCA) cycle and oxidative phosphorylation. This shift may be mediated in part by monocarboxylate transporter 1 (MCT1), which facilitates lactate uptake and enhances metastatic potential [4]. MCT1 inhibition has been shown to reduce metastatic burden by increasing reactive oxygen species (ROS) levels and disrupting oxidative stress management, underscoring the potential influence of metabolic flexibility in melanoma metastasis [4]. Another study revealed a metabolic shift in melanoma metastasis with decreased glycolysis and increased reliance on the TCA cycle [5]. A short isoform of glyceraldehyde‐3‐phosphate dehydrogenase, spermatogenic (GAPDHS), was identified as a key regulator of this switch, with its overexpression suppressing metastasis and its inhibition promoting it through changes in pyruvate carboxylase (PC) activity and aspartate synthesis [5]. One study identified a metabolic shift in melanoma metastasis with decreased glycolysis and increased reliance on the TCA cycle, consistent with evidence that oxidative phosphorylation drives metastatic behavior and therapeutic resistance [6]. Similarly, this study identified that BRAF inhibition in melanoma induces oxidative phosphorylation through upregulation of PGC1α, creating an adaptive metabolic program that limits the efficacy of BRAF‐targeted therapies [6].

Beyond rapid ATP production, glycolysis provides essential intermediates for biomass synthesis, bolstering melanoma cell proliferation. Concurrently, mitochondrial function in melanoma cells is compromised despite functional mitochondria, leading to reduced ATP production and heightened ROS generation [7]. These mitochondrial defects, influenced by mitochondrial DNA mutations and dysregulated nuclear‐encoded genes, contribute to metabolic heterogeneity and enable adaptation to fluctuating environmental stresses [7]. One study evaluated the impact of mitochondrial DNA (mtDNA) mutations in melanoma progression by creating cybrid cell lines with either wild‐type or pathogenic mtDNA variants [8]. While pathogenic mtDNA cybrids established tumors despite impaired oxidative phosphorylation, they disrupted spontaneous metastasis and reduced circulating melanoma cell abundance, indicating that functional mtDNA is favored for metastatic entry into the bloodstream [8]. Another study investigated the effects of truncating mutations in the mtDNA‐encoded complex I gene, Mt‐Nd5, in murine melanoma models, promoting a Warburg‐like metabolic shift that reshaped tumor microenvironments and elicited antitumor immune responses [9]. Tumors with mtDNA mutations showed increased sensitivity to checkpoint blockade therapy, with patient lesions exhibiting over 50% mtDNA mutation heteroplasmy demonstrating a ~ 2.5‐fold improved response rate compared to mtDNA wild‐type tumors, highlighting the potential of mtDNA mutations as functional regulators of cancer metabolism and therapeutic targets [9].

Reliance on mitochondrial function is believed to stem from their enhanced ability to buffer ROS, which represents a significant bottleneck in melanoma metastasis. Redox metabolic rewiring has been implicated in BRAF/MEK‐resistant melanomas [10]. By efficiently managing oxidative stress, melanoma cells increase their resilience and adaptability during the metastatic process, facilitating survival and proliferation in hostile microenvironments. Overexpression of PGC1α (PPARGC1A) in certain human melanomas has been shown to exhibit enhanced mitochondrial metabolism and increased capacity for ROS detoxification, promoting survival under oxidative stress, while PGC1α‐negative melanoma cells demonstrate increased glycolytic capacities and exhibit heightened sensitivity to ROS‐inducing therapies [11]. Glucose 6‐phosphate dehydrogenase (G6PD) mutant melanomas have shown reduced circulating melanoma cells and metastatic burden, exhibiting increased oxidative stress, decreased NADPH, and reliance on malic enzyme activity and glutaminase for compensating oxidative stress, highlighting the layered protective mechanisms conferred by the pentose phosphate pathway, malic enzyme, and glutaminolysis [12]. Successful metastasizing melanomas adapt metabolically to withstand oxidative stress, including increased reliance on NADPH‐generating enzymes in the folate pathway. Antioxidants have been found to promote distant metastasis, while folate pathway inhibition reduces metastatic spread without affecting primary tumor growth [13].

Glycolysis emerges as a central pathway in melanoma, facilitating ATP generation and the synthesis of nucleotides, amino acids, and lipids crucial for cell survival and growth [14]. Lactate, a byproduct of glycolysis, further promotes melanoma progression by signaling angiogenesis and immunosuppression in the tumor microenvironment [14]. Mitochondrial dysfunction complements glycolytic dominance, enabling melanoma cells to thrive amidst therapeutic challenges targeting mitochondrial function. Dysregulated lipid metabolism also plays a pivotal role, supporting tumor growth and metastasis through altered fatty acid synthesis and lipid droplet utilization [15]. Understanding these metabolic intricacies is pivotal for developing targeted therapies that disrupt melanoma metabolism, potentially enhancing treatment efficacy and patient outcomes in cutaneous oncology.

### 3.2 The Tumor Microenvironment and Melanoma Metabolism

The tumor microenvironment (TME) profoundly influences melanoma metabolism, crucially impacting tumor progression, therapeutic responses, and resistance mechanisms [7]. Within the TME, interactions between melanoma cells, stromal components like fibroblasts and endothelial cells, and the extracellular matrix (ECM) drive metabolic adaptations essential for tumor survival and growth [16]. Stromal cells release growth factors, cytokines, and metabolites that influence melanoma metabolism, while ECM components provide structural support and signaling cues. For instance, fibroblasts and endothelial cells enhance glycolysis and lipid metabolism through secreted factors like TGF‐β, HGF, and vascular endothelial growth factor (VEGF), promoting tumor angiogenesis and nutrient supply [17].

Hypoxia, a hallmark of solid tumors including melanoma, triggers adaptive responses in melanoma cells such as upregulating glycolytic enzymes and angiogenic factors mediated by hypoxia‐inducible factors (HIFs) [18]. This metabolic rewiring allows melanoma cells to survive nutrient limitations by increasing autophagy and scavenging nutrients from their environment [19]. Immune cells within the TME also impact melanoma metabolism through paracrine signaling and metabolic competition, influencing tumor progression and therapeutic resistance [20]. For example, immune cells like tumor‐infiltrating lymphocytes (TILs) and tumor‐associated macrophages (TAMs) modulate melanoma metabolism and immune evasion strategies, potentially compromising immunotherapy efficacy [20].

Metabolic adaptations driven by TME interactions contribute significantly to therapy resistance in melanoma [21]. Strategies targeting metabolic dependencies, such as glycolysis or mitochondrial function, may synergize with existing therapies to improve treatment outcomes [21]. Moreover, approaches that modify the TME, including immunomodulatory and anti‐angiogenic therapies, hold promise for sensitizing melanoma cells to metabolic‐targeted treatments [21]. Understanding these complex interactions offers avenues for developing more effective therapeutic strategies tailored to disrupt melanoma metabolism and enhance patient outcomes.

### 3.3 Molecular Mechanisms Driving Metabolic Reprogramming

**FIGURE 2:**
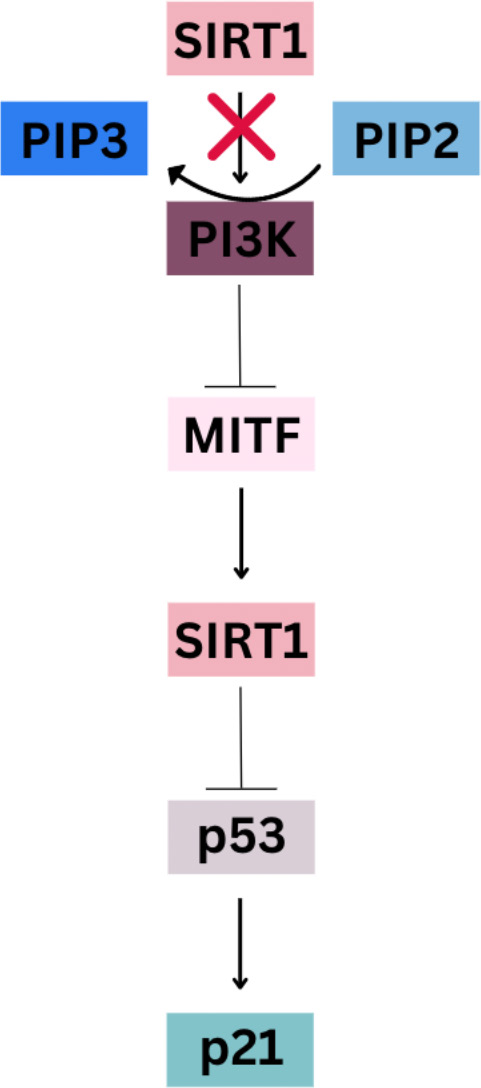
In melanoma cells, SIRT1 inhibition blocks the PI3K‐AKT pathway, removing inhibition of MITF, maintaining p53 acetylation, and interfering with migration.

Recent research highlights the pivotal role of epigenetic modifications in shaping melanoma's metabolic phenotype. DNA methylation, the addition of methyl groups to cytosine residues within CpG dinucleotides, is a crucial epigenetic mechanism regulating gene expression. In melanoma, aberrant DNA methylation patterns are frequently observed and are implicated in the reprogramming of metabolic pathways. Hypermethylation of tumor suppressor genes and hypomethylation of oncogenes contribute to the dysregulation of metabolic genes. Hypermethylation and subsequent silencing of PTEN lead to the activation of the PI3K‐AKT pathway, promoting glucose uptake and glycolysis [22]. Silencing of DNA methyltransferase 1 (DNMT1) enhances the expression of HSPB8 by enhancing its methylation, thereby also reducing binding between HSPB8 and BAG3 [23]. This suggests that DNMT1‐mediated repression of the HSPB8‐BAG3 interaction may activate the PI3K/AKT/mTOR pathway (Figure 2).

In melanoma, histone modifications have been shown to regulate key metabolic pathways. Histone deacetylases (HDACs) [24] can deacetylate HIF‐1α, stabilizing and activating it to promote glycolysis and angiogenesis. Targeting HIFs [25] with drugs can hinder melanoma cell adaptation to hypoxia, reducing glycolytic enzyme and angiogenic factor upregulation, thereby weakening tumor survival in low‐oxygen environments and enhancing susceptibility to other treatments. Inhibiting HDACs can restore oxidative phosphorylation‐related gene expression, shifting metabolism away from glycolysis and potentially sensitizing melanoma cells to metabolic stress.

Sirtuins, NAD + ‐dependent deacetylases, play crucial roles in melanoma. SIRT1 is overexpressed, inhibiting p53‐dependent cell arrest [26] and promoting microphthalmia‐associated transcription factor (MITF)‐induced proliferation [27] (Table 1). SIRT3 enhances mitochondrial pathways and counters oxidative stress‐induced apoptosis [28]. SIRT5 regulates histone modifications, inhibiting melanoma growth when dysfunctional [29]. SIRT6 acts as an oncogene, inducing G1‐phase arrest and senescence‐like phenotypes in melanoma cells [30].

**TABLE 1:** Key metabolic alterations and molecular pathways dysregulated in melanoma.

Mutation/molecular change	Affected pathway	Resultant effect
Hypoxia‐inducible factors (HIFs) [9, 15]	Hypoxia response pathways Histone deacetylation	Activation of genes involved in glycolysis, angiogenesis, and erythropoiesis; adaptation to hypoxic conditions
miR‐211‐5p [21]	Glycolysis promotion	Promotes glycolysis in melanoma cells
miR‐137 [22]	MITF regulation	Downregulates MITF, affecting mitochondrial function and glycolysis
Mitochondrial lncRNAs [23, 24, 25, 26]	Oxidative phosphorylation and glycolysis	Interact with chromatin‐modifying enzymes; repress metabolic genes involved in oxidative phosphorylation; and promote glycolytic metabolism
Nutrient limitation in TME [9]	Metabolic rewiring	Increased autophagy and macropinocytosis for nutrient scavenging
Cytokines (e.g., IFN‐γ, IL‐2) from tumor‐infiltrating lymphocytes (TILs) and tumor‐associated macrophages (TAMs) [10]	Modulation of metabolism and immune evasion; support of tumor progression	Alter melanoma cell metabolism; modulate immune evasion strategies; and promote metabolic adaptation and survival of melanoma cells
Lactate and adenosine production [9]	Immunosuppression	Impairs the function of antitumor immune cells
Sirtuins [17, 18, 19, 20]	Inhibition of p53‐dependent cell arrest; reduction of oxidative stress; regulation of histone modifications for growth inhibition	Promotes cell proliferation and mitochondrial function and enhances cell survival

Non‐coding RNAs, including microRNAs (miRNAs) and long non‐coding RNAs (lncRNAs), regulate gene expression and metabolic pathways in melanoma. MiRNAs like miR‐211‐5p‐5p promote glycolysis in melanoma cells [31], while others are involved in MITF downregulation, such as miR‐137 [31]. Mitochondrial lncRNAs interact with chromatin‐modifying enzymes, affecting metabolic gene expression. SAMMSON [32, 33], RMRP [34], and LINC00473 [35] regulate mitochondrial homeostasis and energy balance, influencing melanoma cell metabolism toward glycolysis.

### 3.4 Therapeutic Implications

Given the central role of epigenetic modifications in melanoma metabolism, targeting these regulators presents a promising therapeutic strategy. Precision medicine approaches leverage these epigenetic markers to stratify patients and select the most effective metabolic therapies.

Epigenetic drugs, such as DNA methyltransferase inhibitors (DNMTis) and HDAC inhibitors (HDACis), have shown potential in preclinical and clinical studies (Table 2). DNMTis, such as decitabine [36], can demethylate and reactivate tumor suppressor genes, thereby inhibiting glycolysis and promoting oxidative phosphorylation. HDACis, such as vorinostat and romidepsin, have been historically able to repress metabolic genes and sensitize melanoma cells to metabolic stress and apoptosis. Studies also show that HDACi may sensitize melanoma cells to immunotherapy and targeted therapy and hence may be combined with immune checkpoint blockade or BRAF and MEK inhibition [37].

**TABLE 2:** Therapeutic targets and associated agents in melanoma treatment.

Treatment	Target
Decitabine [27]	DNA methyltransferases (DNMTs)
Vorinostat [28]	Histone deacetylases (HDACs)
Bevacizumab [35]	Vascular endothelial growth factor (VEGF)
Ramucirumab [36]	Vascular endothelial growth factor (VEGF)
Small molecule tyosine kinase inhibitors (e.g., imatinib, apatinib) [37, 38, 39]	VEGF receptors (VEGFRs)
Sorafenib [41]	RAF/MEK/ERK pathway, VEGF receptors (VEGFRs)
Tenovin‐1 [16], tenovin‐6 [29], sirtinol [30], ex‐527 [30], 4’‐bromo‐resveratrol [31]	Sirtuin deacetylases

Sirtuins have been serving as novel therapeutic targets in this category. Inhibitors of different sirtuins, such as tenovin‐1 [26], tenovin‐6 [38], sirtinol [39], ex‐527 [40], and 4’‐bromo‐resveratrol [41], may result in a significant decrease in cell growth and cell viability, especially in combinations. Decreases in lactate production, glucose uptake, and NAD+ /NADH ratio have been seen with these therapies.

MiRNA mimics and antagomirs represent innovative strategies to manipulate metabolic gene expression in melanoma. While immune responses have been observed [42], enhancing the efficacy of miRNA therapeutics remains crucial. AntagomiR‐221 and antagomiR‐222 show promise due to their effectiveness, stability, and low toxicity at small doses [42]. Researchers are investigating nanoparticle‐based delivery systems and chemical modifications to miRNAs to enhance stability and specificity [43]. Achieving a balance between therapeutic benefits and immune response management is essential for advancing miRNA‐based treatments in melanoma.

Melanoma cells induce angiogenesis to support their metabolic demands, becoming more aggressive and resistant to treatment. VEGF inhibitors like bevacizumab [44] and ramucirumab [45] normalize blood vessels, enhancing oxygen delivery and potentially reducing the tumor's support for cancer cell survival. Resistance to these therapies prompts combination approaches and predictive strategies. Small molecule inhibitors such as axitinib [46], imatinib [47], and apatinib [48] target VEGF receptors (VEGFRs) on endothelial cells, while sorafenib [49] inhibits VEGFRs and RAF/MEK/ERK pathways, serving dual roles as anti‐angiogenic and signaling pathway inhibitors in melanoma therapy.

Targeting metabolic pathways in melanoma is crucial for overcoming therapeutic resistance, particularly in MAPK pathway inhibitor (MAPKi)‐resistant BRAF‐mutant tumors. One study evaluated IACS‐010759 (OPi), a mitochondrial oxidative phosphorylation complex I inhibitor, against MAPKi‐resistant BRAF‐mutant melanomas, demonstrating significant antitumor activity in vitro and in vivo [50]. OPi inhibited oxidative phosphorylation and tumor growth, correlating with decreased MAPK and mTOR complex I activity, increased glucose incorporation into glycolysis, reduced mitochondrial TCA cycle activity, and diminished cellular nucleotide and amino acid pools, highlighting its potential as a therapeutic strategy to overcome intrinsic and acquired resistance to MAPKi [50].

Melanoma is also significantly influenced by ROS, which plays critical roles in cell proliferation, DNA damage, invasion, and drug resistance. While targeting ROS with antioxidants like N‐acetyl cysteine (NAC) has been proposed as a potential strategy for melanoma prevention, the outcomes have been mixed. Clinical studies have shown that NAC can protect against UV‐induced oxidative stress in human nevi, suggesting a preventive role [51]. However, mouse studies indicate that NAC may promote lymph node metastasis without affecting proliferation [52]. Emerging evidence also suggests that targeting oxidative stress‐related pathways, such as those involving NOX and NOS enzymes, may provide new therapeutic avenues. However, the complexity of redox biology in melanoma necessitates a careful approach to antioxidant use, as both dosage and context significantly influence therapeutic outcomes [53]. Overall, while ROS modulation presents a viable strategy in melanoma treatment, a nuanced understanding of redox balance and the specific roles of antioxidants is crucial for developing effective therapies.

Dietary interventions have emerged as a promising strategy to influence the response to melanoma therapy, with significant evidence supporting the role of specific diets in modulating tumor growth and immune response. For instance, a high‐fat ketogenic diet has been found to enhance BRAF V600E mutant‐dependent MEK1 activation, leading to increased tumor growth in BRAF V600E‐expressing melanoma cells [54]. Conversely, ketogenic diets have demonstrated antitumor effects across various melanoma genotypes, reducing tumor growth and metastasis while altering metabolic pathways, including amino acid metabolism [55]. Additionally, dietary fiber intake has been linked to improved progression‐free survival in melanoma patients receiving immune checkpoint blockade (ICB) therapy, indicating the potential impact of gut microbiota interactions [55].

Moreover, targeting specific metabolic pathways through diet has shown promise in combination with pharmacological interventions. A study identified that a low‐oleic acid diet, when combined with an SCD inhibitor, effectively suppressed tumor growth and metastasis in PTEN wild‐type melanoma models while enhancing the efficacy of anti‐PD‐1 immunotherapy [56]. Different diet patterns mold the gut microbiome functional and taxonomic composition as well, and hence, dietary modulation in combination with these effects may also modify melanoma outcomes [57]. Collectively, these findings suggest that dietary modifications can be tailored to an individual's genetic and metabolic tumor profile, potentially enhancing the effectiveness of melanoma treatments and offering new avenues for precision nutrition in cancer therapy. Interdisciplinary and personalized treatment plans hence may be utilized for melanoma patients.

Identifying dysfunction and dysregulation in melanoma via biomarkers enables precise patient stratification and treatment monitoring, enhancing therapeutic outcomes. Personalized medicine shows promise in improving melanoma treatment by addressing epigenetic alterations, metabolic dysregulation, and immune evasion in the tumor microenvironment.

## 4 Conclusion

This review underscores the profound metabolic reprogramming in melanoma, highlighting increased glycolysis, altered mitochondrial function, and enhanced lipid metabolism as pivotal to its pathogenesis. These changes support rapid cell growth and survival: heightened glycolysis via the Warburg effect enables quick ATP generation and biosynthetic intermediates production, while altered mitochondria function, alongside oxidative phosphorylation maintenance, increases ROS production and enhances metabolic flexibility under stress. Elevated lipid metabolism, encompassing de novo lipogenesis and fatty acid oxidation, supplies critical components for membrane synthesis and energy storage, further driving tumor progression. The tumor microenvironment (TME) and epigenetic mutations profoundly influence these pathways, with hypoxia and nutrient scarcity in the TME triggering adaptive responses like autophagy and macropinocytosis. Future research should elucidate these mechanisms to identify therapeutic targets and biomarkers for tailored treatments in melanoma. Targeting these metabolic vulnerabilities, possibly through glycolytic enzyme inhibition or mitochondrial and lipid pathway modulation, in combination with current therapies, could enhance treatment efficacy and overcome resistance. Understanding melanoma metabolism promises innovative strategies for personalized oncology, potentially transforming patient outcomes and treatment paradigms.

## Author Contributions

**Isabella J. Tan:** conceptualization (lead), data curation (equal), methodology (equal), writing – original draft (equal), writing – review and editing (equal). **Aarushi K. Parikh:** conceptualization (equal), data curation (equal), methodology (equal), writing – original draft (equal), writing – review and editing (equal). **Bernard A. Cohen:** supervision (lead), writing – review and editing (equal).

## Ethics Statement

The authors have nothing to report.

## Conflicts of Interest

The authors declare no conflicts of interest.

## Data Availability

Data sharing is not applicable to this article as no new data were created or analyzed in this study.
